# Supplementary material for: Content-rich biological network constructed by mining PubMed abstracts
Source: BMC Bioinformatics. 2004 Oct 8;5:147. doi: 10.1186/1471-2105-5-147 (PMC528731; doi:10.1186/1471-2105-5-147)
Supplement: Additional File 2 — The original results of the above study (non-essential files are deleted to keep the file size under the limit set by BMC bioinformatics). [file 1471-2105-5-147-S2.bz2 › chilibotAdditionalFile2/dip20/603ID9751050E10982/html/CDK6_CDKN2D.html]

 


 **CDK6** and **CDKN2D** 
  
Found 24 abstracts in PubMed, retrieved 20.  
 

 What does Google say? 
 PDF only 
| .edu only 

---

**Interactive relationship** (e.g. stimulation, inhibition, etc)

**Inhibitory relationship**- Crystal structure of the complex of the cyclin D dependent kinase  **Cdk6**  bound to the cell cycle inhibitor p19INK4d  [ **CDKN2D** ] .  Ref: 9751051 Nature, 1998
- The INK4 family of cyclin dependent kinase CDK inhibitors includes four 15 to 19 kDa polypeptides p16 INK4a, p15 INK4b, p18 INK4c, and p19 INK4d  [ **CDKN2D** ]  that bind to CDK4 and  **CDK6** .  Ref: 10594039 Mol Cell Biol, 2000
- While members of the INK4 family p16INK4A, p15INK4B, p18INK4C, p19INK4D  [ **CDKN2D** ]  interact specifically with CDK4 and  **CDK6** , the CIP KIP inhibitors p21CIP1 WAF1, p27KIP1 and p57KIP2 inhibit a broader spectrum of CDK.  Ref: 11355576 Cancer Radiother, 2001
- The structures of  **Cdk6**  bound to p16INK4a and to the related p19INK4d  [ **CDKN2D** ]  reveal that the INK4 inhibitors bind next to the ATP binding site of the catalytic cleft, opposite where the activating cyclin subunit binds.  Ref: 9751050 Nature, 1998
- We also review our recent structural studies on intracellular signalling complexes, focusing on phosducin transducin GPry, CK2 protein kinase and its complexes, and the cyclin D dependent kinase,  **Cdk6** , bound to the cell cycle inhibitor p19INK4d  [ **CDKN2D** ] .  Ref: 11076027 Biol Chem< MedlineTA>Biol Chem, 1998
- BACKGROUND The four members of the INK4 gene family p16 INK4a, p15 INK4b, p18 INK4c and p19 INK4d  [ **CDKN2D** ]  inhibit the closely related cyclin dependent kinases CDK4 and  **CDK6**  as part of the regulation of the G1 >S transition in the cell division cycle.  Ref: 9782052 Structure, 1998
- p19INK4d  [ **CDKN2D** ]  is a 165 amino acid protein that belongs to the INK4 family of CDK4 and  **CDK6**  inhibitors.  Ref: 9013872 FEBS Lett, 1997
- The four members of the INK4 gene family, p16 INK4a, p15 INK4b, p18 INK4c and p19 INK4d  [ **CDKN2D** ] , are known to bind to and inhibit the closely related cyclin dependent kinases CDK4 and  **CDK6**  as part of the regulation of the G1 S transition in the cell division cycle.  Ref: 9761685 J Mol Biol, 1998
**Neutral relationship**- d most importantly, the extra loop of p18 appears to contribute to the function of p18, even though the crystal structure of the p19INK4D  [ **CDKN2D** ]   **CDK6**  complex indicates no interactions involving this loop.  Ref: 10651629 Biochemistry, 2000
- The 50 70 kDa complex was made up of two moieties, a 66 kDa complex containing  **cdk6**  bound to p19INK4d  [ **CDKN2D** ]  and monomeric  **cdk6** .  Ref: 9482106 Oncogene, 1998
- The p19INK4d  [ **CDKN2D** ]  and p18INK4c proteins formed complexes with either CDK4 or  **CDK6**  in a temporal pattern consistent with the results of in situ hybridization.  Ref: 9372237 Cell Growth Differ, 1997
- A possible mode of binding between p19 INK4d  [ **CDKN2D** ]  and CDK4 and  **CDK6**  could therefore involve the loop segments of p19 INK4d  [ **CDKN2D** ] .  Ref: 9761685 J Mol Biol, 1998
- The kinase activation was found to result from Tax induced expression of genes for cell cycle regulatory molecules including cyclin D2, cyclin E, E2F1, CDK2, CDK4 and  **CDK6** , and Tax induced reduction of CDK inhibitors p19 INK4d  [ **CDKN2D** ]  and p27 Kip1.  Ref: 11360190 Oncogene, 2001
- P19 INK4d  [ **CDKN2D** ]  is a tumor suppressing protein and belongs to a family of cyclin D dependent kinase inhibitors of CDK4 and  **CDK6** , which play a key role in human cell cycle control.  Ref: 11786024 J Mol Biol, 2002

**Non-interactive relationship** (e.g. studied together, co-existance, homology, etc.)

- The crystal structure of the cyclin D dependent kinase  **Cdk6**  bound to the p19 INK4d  [ **CDKN2D** ]  protein has been determined at 1.9 A resolution.  Ref: 9751051 Nature, 1998
- In situ hybridization of mouse embryo sections demonstrated expression of mRNAs encoding two polypeptide inhibitors p18INK4c and p19INK4d  [ **CDKN2D** ]  of cyclin D dependent kinase CDK 4 and  **CDK6**  in the central nervous system.  Ref: 9372237 Cell Growth Differ, 1997
- Expression of p19INK4d  [ **CDKN2D** ] , CDK4,  **CDK6**  in glioblastoma multiforme.  Ref: 10884881 Br J Neurosurg, 2000
- We derived 32D cells in which the expression of p19INK4d  [ **CDKN2D** ] , a specific polypeptide inhibitor of CDK4 and  **CDK6** , is regulated by the heavy metal inducible sheep metallothionein promoter.  Ref: 9207446 Blood, 1997
- Analysis of normal B cell precursors by Western blotting indicated that CDK4,  **CDK6** , p19 INK4d  [ **CDKN2D** ] , and p57 KIP2 were expressed.  Ref: 11301189 Exp Hematol, 2001
